# Adherence and health beliefs in a psychoeducative intervention in naïve HIV-1-infected men: the PROADH study

**DOI:** 10.7448/IAS.15.6.18092

**Published:** 2012-11-11

**Authors:** C Fumaz, J Muñoz-Moreno, M Ferrer, M Gonzalez-Garcia, N Perez-Alvarez, C Miranda, E Negredo, B Clotet

**Affiliations:** 1 HIV Unit, Germans Trias i Pujol University Hospital, Lluita contra la SIDA Foundation, Barcelona, Spain; 2 HIV Unit, Lluita contra la SIDA Foundation, Barcelona, Spain; 3 HIV Unit, HIVACAT, IrsiCaixa Foundation, Barcelona, Spain

## Abstract

**Background:**

Health beliefs are an important factor in the maintenance of an adherent behaviour. However, specific interventions based on the modification of health beliefs to promote adherence have not been applied in naïve HIV-infected subjects.

**Methods:**

Prospective randomized 48-week study to evaluate the efficacy of a psychoeducative intervention based on health beliefs to promote adherence in a sample of naïve HIV-1-infected men who started antiretroviral therapy. Participants were randomized to follow three intervention visits to promote adherence with the use of projective drawing techniques, Life-steps and Motivational interview (Intervention Group; GI) or to continue with the routine care (Control Group; GC). Adherence was assessed through self-report and drug plasma levels. Mann-Whitney nonparametric test, w2 or Fisher exact test were used to compare variables.

**Results:**

Participants were 40 men with a median (IQ) age of 35.2 (30.2–44.8) years, CD4 cell count at the study entry of 316 (229–539) cells/mm^3^ and HIV-RNA VL of 65.000 (22.500–250.000) copies. The infection route had been mainly MSM (90%). QD and BID ARV therapy was prescribed in 29 (72.5%) and 11 (27.5%) subjects. Seven patients (2 in GI; 5 in GC) were lost to follow-up. At week 48, 100% of subjects in GI and 60% in GC had 100% adherence (p=0.01). In GC, 26% and 14% of subjects had ≥95% and <95% adherence, respectively. No differences were found in adherence regarding QD or BID therapy. All subjects except for 3 had VL <25 copies at week 48.

**Conclusions:**

High adherence was observed in the majority of this group of naïve HIV-infected men who initiated their first antiretroviral therapy. However, all subjects following the intervention had 100% adherence after one year of follow-up. A psychoeducative intervention based on the modification of health beliefs may be a useful strategy to promote adherence in naïve HIV-infected patients.

